# The episodic buffer in children with intellectual disabilities: An exploratory study

**DOI:** 10.1016/j.ridd.2010.04.025

**Published:** 2010-11

**Authors:** Lucy A. Henry

**Affiliations:** Department of Psychology, London South Bank University, 103, Borough Road, London SE1 0AA, UK

**Keywords:** Episodic buffer, Children, Intellectual disabilities, Working memory

## Abstract

Performance on three verbal measures (story recall, paired associated learning, category fluency) designed to assess the integration of long-term semantic and linguistic knowledge, phonological working memory and executive resources within the proposed ‘episodic buffer’ of working memory (Baddeley, 2007) was assessed in children with intellectual disabilities (ID). It was hypothesised that children with ID would show equivalent performance to typically developing children of the same mental age. This prediction was based on the hypothesis that, despite poorer phonological short-term memory than mental age matched peers, those with ID may benefit from more elaborate long-term memory representations, because of greater life experience. Children with ID were as able as mental age matched peers to remember stories, associate pairs of words together and generate appropriate items in a category fluency task. Performance did not, however, reach chronological age level on any of the tasks. The results suggest children with ID perform at mental age level on verbal ‘episodic buffer’ tasks, which require integration of information from difference sources, supporting a ‘delayed’ rather than ‘different’ view of their development.

The episodic buffer of working memory (Baddeley, 2000; Baddeley, 2007) is proposed as a limited capacity storage system responsible for integrating information from several sources to create a unified memory, sometimes referred to as a single ‘episode’. The episodic buffer does this by “binding” information from the various systems of working memory (e.g. phonological loop, visuospatial sketch pad) and relevant activated long-term semantic and linguistic knowledge, into a coherent whole. The episodic buffer can also act as a “mental modelling space, allowing one to set up representations that might guide future actions” (Baddeley & Wilson, 2002, p. 1738).

The current paper is concerned with the integration/binding of linguistic and semantic long-term knowledge with information from the “slave” verbal storage system, the phonological loop, during verbal episodic remembering and thinking tasks. It provides an exploratory investigation of whether the functioning of the episodic buffer in children with intellectual disabilities (IQ below 70, associated adaptive and daily living difficulties) is at a level commensurate with their mental age. Children with ID were compared to comparison groups of typically developing children matched for chronological and mental age. Such groups allow the evaluation of three competing cognitive accounts of ID: (1) according to the ‘developmental’ model, children with mixed aetiology familial ID should obtain mental age-appropriate levels of performance on cognitive tasks, as their cognitive development is delayed rather than different in comparison with typical controls (Zigler, 1969); (2) evidence for below mental age level performance on cognitive tasks supports the ‘difference’ model, that cognitive development in those with ID is different to those with typical development (Ellis, 1969; Milgram, 1973); and (3) evidence for ‘optimal performance’ is found when levels of performance in those with ID approach chronological age level (Burack & Zigler, 1990).

A small literature is developing on the episodic buffer in typically developing children. For example, it has been claimed that the episodic buffer is a separate component of working memory that can be reliably identified. Alloway, Gathercole, Willis, and Adams (2004) used ‘recall of spoken sentences’ to assess integration of phonological short-term memory representations with relevant long-term language knowledge. They argued that this measure tapped a distinct component of working memory in 4–6-year-old children (i.e. the episodic buffer), which was separable from the phonological loop and the central executive; although the three components were somewhat related. Others have suggested that the proposed *automatic* binding mechanisms in the episodic buffer, responsible for integrating information from different sources, may mature relatively early in typically developing children (possibly as young as 6 years, Sluzenski, Newcombe, & Kovacs, 2006). Experimental work in support of this conclusion has concerned tasks such as the binding of visual object and spatial information (Cowan, Naveh-Benjamin, Kilb, & Saults, 2006), verbal paired associate recognition (Shing, Werkle-Bergner, Li, & Lindenberger, 2008), and memory for combinations of complex photographs of animals in variable backgrounds (Sluzenski et al., 2006). There is also evidence that different tasks assessing episodic buffer functioning may assess common processes. Sluzenski et al. (2006) looked at the relationship between picture association and story remembering, finding modest correlations between the two tasks (about .40). This could be interpreted as evidence in favour of a general episodic buffer component which binds information from different sources together, regardless of exact content.

Although many authors investigating memory performance generally in children with ID (Weiss, Weisz, & Bromfield, 1986) and phonological short-term memory, in particular, have reported *below mental age level* abilities (Henry & MacLean, 2002; Henry & Winfield, 2010; Hulme & Mackenzie, 1992; Van der Molen, Van Luit, Jongmans, & Van der Molan, 2007), episodic buffer functioning may be *as good as* children matched for mental age in children with ID. If automatic binding processes mature early in typically developing children, this implies that these skills are not greatly influenced by developmental factors and may be relatively spared in children with ID. Perhaps more importantly, children with ID are likely to have *greater* levels of semantic and linguistic long-term knowledge to support episodic remembering, than children matched for mental age, who are necessarily younger and have less life experience. It is proposed that greater long-term knowledge might compensate children with ID for their weaker phonological short-term memory on tasks which require the integration of information from both of these sources in the proposed episodic buffer of working memory. There is some corroborating evidence that the knowledge base of individuals with ID is more extensive than that of typically developing mental age matched controls (Lukose, 1987; Numminen, Service, & Ruoppila, 2002).

Therefore, three verbal recall/thinking tasks, hypothesised to require the binding of information from phonological short-term memory with activated semantic and linguistic knowledge from long-term memory were employed in the present study. All tasks were necessarily indirect measures of the proposed episodic buffer, as no clearly agreed methodologies exist for assessing the episodic buffer independently.

The first task was *prose recall*. In order to recall a passage of text coherently, it is necessary to utilise long-term knowledge about the structure of language, vocabulary, content of the passage and the structure of typical narratives or scripts. This information must be integrated with memory traces from the phonological loop, and “modality free” representations held in the episodic buffer. Storage in the phonological loop alone is insufficient to support prose recall. The episodic buffer is hypothesised to create a “novel episode”, by combining primed or activated representations from long-term memory with information in the phonological loop, drawing on executive resources to maintain this new representation (Baddeley & Wilson, 2002). Therefore, all participants recalled short, but coherent, stories from a standardised children's memory battery (Test of Memory and Learning, TOMAL, Reynolds & Bigler, 1994). This immediate story recall task is directly analogous to that used by Baddeley and Wilson (2002) in their study of densely amnesic patients (i.e. ‘logical memory’ from the Wechsler Memory Scale).

The second task was *paired associate learning* from the TOMAL (Reynolds & Bigler, 1994). Participants were asked to learn to associate pairs of words over several trials (e.g. bite-name); and half of the items were already associated (e.g. stove-cook), so long-term knowledge should have been even more likely to aid learning. Performance on this task can be hypothesised to reflect the capacity of the phonological store, activated long-term knowledge about the meanings of words and potential associations between them, the quality of the integrated “novel episode” created by the episodic buffer, and the efficiency the relevant executive processes recruited to maintain this representation. Some earlier papers on paired associate learning in ID provide relevant background to the current study. For example, Winters, Attlee, and Harvey (1974) found that teenagers with ID (provided they were non-institutionalised) were as able to use long-term knowledge as typically developing children of the same mental age in picture paired associate task (see also Cantor & Ryan, 1962). However, the IQs of participants in the Winters et al. (1974) study were only assessed with a receptive vocabulary test, and those with IQs over 70 do not appear to have been excluded. The current study employed two of the three full subscales from the British Ability Scales II (Elliott, 1996) to obtain more reliable estimates of IQ, and only included children with ID who had IQs of 70 and below.

The final task was category fluency. Fluency or ‘generation’ tasks are often discussed in the context of executive processing (e.g. Bishop & Norbury, 2005), but in addition to requiring executive skills to coordinate the search for relevant exemplars and inhibit items that have already been selected, category fluency relies on lexical access: specifically, accessing stored long-term semantic knowledge about which items belong to particular categories (Fisk & Sharp, 2004). Category fluency, therefore, may be a good example of the interface between central executive resources and links to semantic memory via the episodic buffer. Existing evidence suggests that verbal fluency may be at mental age level in those with ID, but much of this research is somewhat inconclusive due to the absence of mental age (Glidden & Mar, 1978) or chronological age comparison groups (Conners, Carr, & Willis, 1998); or the inclusion of individuals with borderline ID (Van der Molen et al., 2007).

In summary, this study explored the performance of children with ID on a series of tasks hypothesised to assess the proposed episodic buffer of working memory, and compared them to comparison groups of similar chronological and mental age. All tasks were hypothesised to require the “binding” of information from different sources (long-term memory and phonological short-term memory). It was predicted that although children with ID may have a *disadvantage* with respect to phonological short-term memory compared to mental age matched peers, they were likely to have an *advantage* in terms of long-term semantic and linguistic knowledge. The possibility of greater support from long-term memory was, therefore, predicted to compensate for poorer phonological short-term memory on the three verbal tasks used in the current study. In sum, the hypothesis was that children with ID would obtain overall scores *on a par with* their mental age matched peers, supporting the developmental model.

## 1 Method

### 1.1 Design

This study employed a between subjects design with three groups of children: 11–12-year-old children with mild and moderate ID; 11–12-year-old typically developing children (CA comparison group); and 5–8-year-old typically developing children with mental ages comparable to those with ID (MA comparison group). These children participated in an earlier study of eyewitness memory (Henry & Gudjonsson, 2003), but the criteria for selection were slightly more stringent in the current study as follows: those with IQs over 70 in the ID group (*n* = 8), and those with mental ages above 110 months in the MA group (*n* = 3) were not included. Apart from these exclusions, every participant from the earlier study was included. All participants were assessed with the Verbal Reasoning (word definitions, verbal similarities) and Non-Verbal Reasoning (matrices, quantitative reasoning) scales from the British Ability Scales II (Elliott, 1996) to provide an estimate of IQ.

As part of their assessment in our earlier study, all children were administered the Test of Memory and Learning (TOMAL, Reynolds & Bigler, 1994) and a category fluency measure, but detailed data from these tests has not been reported until now. Written informed consent was obtained from the parents of all participants, and the research study was approved by the Ethics Committee of the Institute of Psychiatry, Kings College London.

### 1.2 Participants

Table 1 includes full details of all participants. Thirty-nine children (mean age in ‘years:months’ of 11:11) with non-specific aetiology mild and moderate ID participated in this study (mean IQ = 54.5). The majority of participants attended special secondary schools for children with ID and had, therefore, been assessed with both ID and further adaptive difficulties. A small additional number of children were recruited from mainstream schools and these individuals were identified by special needs teachers as having marked learning and support needs. Although data on aetiology were not collected, individuals with known developmental disorders such as autism or Down syndrome were not included.

The chronological age comparison group (CA) included 25 11–12-year-old typically developing children (mean age 11:11), selected from mainstream schools and with no special needs identified (mean IQ = 104.5). The mental age comparison group (MA) included 25 5–8-year-old typically developing children (mean age 7:1), selected from mainstream schools with no special needs identified (mean IQ = 101.8). Note that for the three youngest children in the MA group, mental age could be calculated, but not IQ, as the BAS II test does not provide norms below the chronological age of 6 years zero months; therefore, the IQ data given in Table 1 excludes these three participants.

The mental age levels for the ID and MA groups were matched as closely as possible at the group level: mean mental age in years and months was 7:2 for the ID group; and 7:4 for the MA group. Mean chronological ages for the ID and CA comparison groups were also closely matched at the group level (11:11 in both cases). See Table 1 for further details. Three missing data points that arose to due time constraints in testing during a busy school timetable were replaced with the series mean, a conservative approach unlikely to affect the results. These included two scores from paired recall and one score from memory for stories in the ID group (Field, 2005).

### 1.3 Procedure

Children were seen in four sessions at their schools (see Henry & Gudjonsson, 2003, for full details), and tests relevant to this study were administered in sessions 3 and 4. These included four subtests from the British Ability Scales II, and six subtests from the TOMAL (Reynolds & Bigler, 1994).

The two tasks from the TOMAL that were of interest here were as follows. First, *memory for stories*, which involved the child hearing three short paragraphs and recalling as much information as possible from them (as per standardised instructions, stories appropriate to age level were administered and the immediate recall condition only was used). Second, *paired recall* which involved the child attempting to learn pairs of words in a cued recall procedure (the number of pairs of words was administered according to age as per standardised instructions and included six pairs for those in the MA group and eight pairs for those in the CA and ID groups) over six learning-and-recall trials. The first word of each pair was used as a cue for recall of the second word (e.g. “bite-*name*”; “stove-*cook*”). Half of the word pairs included related items and half included unrelated items. For both tasks, raw scores, representing the total number of story units recalled over the three stories and the total number of words recalled, were used as the dependent measure of performance. It was important to use raw scores, because age-scaled measures would not reflect absolute levels of performance, which were of interest in comparing the performance of those with ID to the two comparison groups. Note that the ID group had the potential to obtain higher raw scores than the MA group, because they were administered slightly longer stories and slightly more pairs of words (as per standardised instructions). However, this should act against our hypothesis that the ID and MA groups will not differ from each other. It was not considered appropriate to administer MA-level items to the ID group, because this would have violated the standardisation of the test.

The final task was *category fluency*, included in the original study as a pilot measure. The category fluency task involved asking each child to generate as many items from a semantic category as possible in a 1-min period. The task was repeated twice, once for ‘animals’ and once for ‘food items’; and the order of receiving these two semantic categories was counterbalanced across participants. This task was very similar in structure to other well-respected category fluency measures (e.g. Delis-Kaplan Executive Function System, Delis, Kaplan, & Kramer, 2001). Scores represented the number of discrete and different items correctly generated from the relevant category within the time limit; repeated or highly similar items were not scored (e.g. ‘dog’, ‘doggie’). The reliability of this measure was assessed by comparing total scores for animals and foods; Cronbach's alpha was .76, indicating acceptable reliability. For analysis, combined fluency scores on both measures were used.

## 2 Results

Table 1 includes mean scores, SDs and ranges for each of the three episodic buffer tasks, for children with ID and those in the MA and CA comparison groups. Data for memory for stories and category generation were normally distributed without excessive skewness or kurtosis. Data for paired recall showed a slight tendency towards negative skewness (*z* = 1.98), which was so close to acceptable limits (Field, 2005) that parametric statistics were used. There was no evidence for outliers, floor or ceiling effects in any of the groups and the ranges of scores were broadly comparable across groups.

Univariate analyses of variance were used to explore group differences on each of the three tasks (memory for stories, paired recall, category generation). The analysis on memory for stories, revealed a significant effect of group, *F*(2, 86) = 35.73, *p* < .001, *partial η*^2^ = .45. Post hoc Bonferroni tests indicated that those in the CA group obtained significantly higher scores than those in either the ID (*p* < .001, *r* = .71) or MA (*p* < .001, *r* = .70) groups, but the ID and MA groups did not differ (*r* = .06).

Group differences were also significant for paired recall, *F*(2, 86) = 20.72, *p* < .001, *partial η*^2^ = .33. Post hoc Games–Howell tests (equal variances not assumed) indicated that those in the CA group obtained significantly higher scores than those in either the ID (*p* < .001, *r* = .58) or MA (*p* < .001, *r* = .64) groups, but the ID and MA groups did not differ (*r* = .09).

Similarly, there was a significant group difference on category generation, *F*(2, 86) = 18.46, *p* < .001, *partial η*^2^ = .30. Post hoc Bonferroni tests indicated that those in the CA group obtained significantly higher scores than those in either the ID (*p* < .001, *r* = .49) or MA (*p* < .001, *r* = .62) groups, but the ID and MA groups did not differ (*r* = .25).

## 3 Discussion

The purpose of this study was to present an exploratory analysis of the functioning of the proposed episodic buffer of working memory among children with ID, comparing them to typically developing peers matched for mental and chronological age. Children performed verbal memory and thinking tasks that required the integration of long-term semantic and linguistic knowledge with information stored in phonological short-term memory, to create novel episodic records or generate exemplars. It was predicted that children with ID would be as able as MA peers to remember details from a short story, learn to associate pairs of words over several learning trials and generate items from named semantic categories. In line with predictions, children with ID did not differ from MA peers on any task, supporting the predictions and also the developmental hypothesis (Zigler, 1969) of ‘delayed’ rather than ‘different’ development in the episodic buffer in this population. Performance did not reach chronological age levels, offering no support for an optimal performance position.

Children with ID did not have difficulties over and above their general cognitive level (mental age) in binding information from long-term memory together with information from other sources such as the phonological loop, and maintaining and recalling these novel representations (note that Baddeley & Wilson, 2002, suggest maintenance requires executive resources). It was suggested in the introduction that, given their documented difficulties with phonological short-term memory (e.g. Henry & MacLean, 2002; Hulme & Mackenzie, 1992), children with ID (who are older than mental age peers) may benefit from more elaborate long-term semantic and linguistic knowledge in binding tasks that assess the hypothesised episodic buffer. The current results are consistent with this view, although cannot directly assess it.

The present findings are also consistent with previous reports that the performance of individuals with ID reaches mental age level or above, on tasks assessing the use of long-term semantic and linguistic knowledge to support memory, executive functioning and learning (Conners et al., 1998; Lukose, 1987; Van der Molen et al., 2007; Winters et al., 1974). Such findings tie in well with research on eyewitness memory skills in children with ID. On these more naturalistic (usually delayed) memory tasks, integrating information from a range of sources should contribute to producing an episodic record of a witnessed event. Children with ID often reach mental age (e.g. Gordon, Jens, Hollings, & Watson, 1994; Henry & Gudjonsson, 1999, 2003; Michel, Gordon, Ornstein, & Simpson, 2000; although see Agnew & Powell, 2004) or sometimes chronological age (Henry & Gudjonsson, 1999, 2003) levels of recall performance in experimental studies of eyewitness skills.

The clinical implications of these results are that children with ID may show relatively good (i.e. mental age-appropriate) performance on more naturalistic verbal remembering tasks that: (1) draw on long-term knowledge and (2) require the integration of information from different working memory systems such as phonological short-term memory, long-term memory and possibly the hypothesised “modality free” storage capacity of the episodic buffer. Therefore, educational approaches/interventions that present information in several modalities and capitalise on long-term memory knowledge, should be beneficial for children with ID. Such interventions could be used in combination with additional methods to compensate for the weak phonological short-term memory documented in previous studies (Henry & MacLean, 2002; Henry & Winfield, 2010; Hulme & Mackenzie, 1992; Van der Molen et al., 2007). For example, Gathercole, Lamont, and Alloway (2006) suggest the following interventions for children with poor phonological short-term memory: (1) using memory aids/supports (personal boards on desks with key information or visual reminders); (2) reducing verbal demands (very short simple subject-verb-object sentences); and (3) managing processing loads in classroom tasks (reducing vocabulary demands, presenting one task at a time, adopting clear task structures).

A final point to note is that long-term memory *per se* was not directly assessed here, i.e. the ability to store and retain material over long time periods (for an article on long-term memory, see Turnure, 1991, who argues that LTM capacity is largely intact among those with ID). Rather, the focus was on how effectively individuals with ID use the proposed episodic buffer of working memory to integrate relevant, activated long-term knowledge with current working memory processing and storage systems. Additionally, the current work focused on verbal tasks. Paradigms requiring participants to learn colours paired with *nonsense shapes* have revealed deficits relative to mental age among individuals with ID (e.g. Blue, 1970; Vakil, Shelef-Reshef, & Levy-Shiff 1997). Similarly, adults with ID have been reported to show more conjunction errors than controls on a face recognition task utilising combinations of inner and outer details from unfamiliar images (Danielsson et al., 2006). Unfamiliar non-verbal materials may, therefore, present fewer opportunities than familiar verbal materials for integrating long-term semantic and linguistic knowledge, but further research will be necessary to test this speculation.

## 4 Conclusions

The functioning of the proposed episodic buffer of working memory has received relatively little attention. One way of assessing its role in integrating activated long-term memory knowledge with the contents of other working memory systems to support online memory and processing, is to examine tasks such as prose recall, paired recall and category generation. The present findings were that children with ID showed an ability to bind together relevant long-term knowledge and information from short-term phonological memory to produce novel episodic memories and search semantic memory, at a level commensurate with their mental, but not their chronological age. These exploratory results supported the ‘developmental’ model of intellectual disability, that cognitive performance in the episodic buffer is delayed rather than different in this population (e.g. Zigler, 1969).

## Figures and Tables

**Table 1:** Mean values for participant variables and raw scores on episodic buffer tasks are given on each upper line (SD in brackets); with ranges of values/scores on each lower line (in italics).

	ID group *n* = 39	MA group *n* = 25	CA group *n* = 25	Direction of group difference
Chronological age (in years:months)	11:11 (4.7 m) *11:4–12:9*	7:1 (10.2 m) *5:7–8:5*	11:11 (4.3 m) *11:4–12:6*	(ID = CA) > MA
Mental age	7:2 (11.5 m) *5:4–9:0*	7:4 (12.8 m) *5:9–9:2*	12:0 (14.7 m) *10:6–15:0*	(ID = MA) < CA
IQ	54.5 (9.2) *39–70*	104.5 (9.3) *82–124*	101.8 (9.9) *84–123*	ID < (CA = MA)
Memory for stories	23.42 (11.22) *3–58*	24.88 (10.37) *7–52*	45.76 (10.99) *28–72*	(ID = MA) < CA
Paired recall	18.43 (6.89) *1–32*	17.36 (4.47) *7–23*	26.28 (3.86) *16–31*	(ID = MA) < CA
Category generation	28.64 (7.07) *14–45*	24.84 (7.35) *13–43*	37.40 (8.45) *21–51*	(ID = MA) < CA
